# The science of soft robot design: A review of motivations, methods and enabling technologies

**DOI:** 10.3389/frobt.2022.1059026

**Published:** 2023-01-18

**Authors:** Francesco Stella, Josie Hughes

**Affiliations:** CREATE Lab, Institute of Mechanical Engineering, EPFL, Switzerland

**Keywords:** soft robot design, computational design, design methods, bioinspiration and biomimetics, soft robotic technologies

## Abstract

Novel technologies, fabrication methods, controllers and computational methods are rapidly advancing the capabilities of soft robotics. This is creating the need for design techniques and methodologies that are suited for the multi-disciplinary nature of soft robotics. These are needed to provide a formalized and scientific approach to design. In this paper, we formalize the scientific questions driving soft robotic design; what motivates the design of soft robots, and what are the fundamental challenges when designing soft robots? We review current methods and approaches to soft robot design including bio-inspired design, computational design and human-driven design, and highlight the implications that each design methods has on the resulting soft robotic systems. To conclude, we provide an analysis of emerging methods which could assist robot design, and we present a review some of the necessary technologies that may enable these approaches.

## 1 Introduction

Soft robotics has introduced a range of robotic technologies with wide ranging form, function and appearance, with their inherent compliance opening up new application domains for robotics and leading to the creation of novel fundamental technologies (Lipson, 2014; Rus and Tolley, 2015). To date, soft robots have been successful applied to underwater exploration (Katzschmann et al., 2018; Li et al., 2021), rehabilitation robotics (Liu et al., 2020; Ashuri et al., 2020) and manipulation solutions (Hughes et al., 2016) amongst others. This wide varying range of applications has been enabled by the creation of fundamental ‘soft-robotic technologies’ and accompanying control and learning algorithms (Laschi et al., 2016; Della Santina et al., 2021).

The diversity in materials and mechanisms, actuation technologies, sensors and control approaches provides a design space that allows for creative, and innovative solutions. This creativity seen in soft robot design should be celebrated; however, it still falls far short of the diversity and variety that can be found in nature (Fine, 2015). Whilst we need to further extend and search this design space to increase the diversity and creativity of solutions, the possibility of exploring a wide range of solutions leads to a conflicting challenge of isolating or finding optimal design solution. Although there has been significant advances in modelling and representing soft systems, this is still an ongoing and open quest (Schegg and Duriez; 2022). This means that soft robot design relies heavily on human intuition and experience. Although this has been shown to lead to many successful and impact robotic solutions and approaches, these can be challenging to formalize the fundamentals that underpin the development of soft robotic technologies and solutions.

In light of this conflicting needs and challenges for soft robot design, we review the scientific questions driving soft robotic design, and current methods and approaches used for soft robot design. We conclude by providing some potential directions and approaches which would allow us to design increasingly capable soft robotic solutions.

### 1.1 What is the science behind soft robot design?

Soft robotics offers a multi-disciplinary approach, where the behavior can be determined by the robot materials, morphology, control and the interactions with the environment. It also denotes a design problem where the design space or intelligence is distributed between the body-brain-environment interactions (Pfeifer et al., 2007). Although this can be the case (Collins et al., 2001) for ‘rigid’ robots it is less commonly so. Therefore, the design of soft robots can not be limited to a morphological problem, but instead it should consider the entire embodiment in the system, composed of the mechanical structure, the control strategy and the interaction with the environment.

**FIGURE 1:**
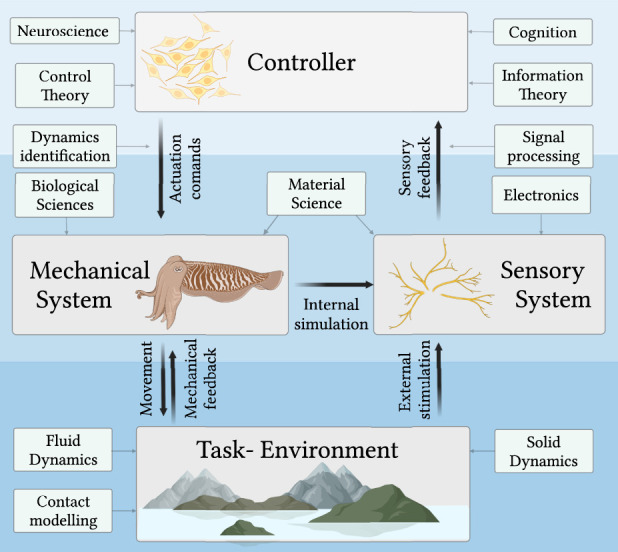
A schematic representation of the disciplines involved in the design of soft robots with embodied intelligence, i.e., systems in which the intelligence spans between Brain, Body and environmental interaction.

As shown in Figure 1 soft robotic systems exploit the interactions between the physicality of the structure, traditional computation and also the environment. In this way, we have physical interactions between the soft body and the environment. Thus to be able to *explain* and hence *design* the resultant behaviors of soft robotic systems, many different scientific disciplines are required. In addition to the physics and science governing the behaviors of soft or even biological materials, we also require an understanding of the physical interactions between the soft body and the environment. This can require insight into fluid dynamics, mechanics of solids, or even soil dynamics.

To develop design methodologies there is a requirement to understand or model many different scientific disciplines, and also their interactions. For example, to understand how the sensory cognition and control of a soft structure affects the resultant fluid dynamics when it is moving in a water based environment, a synergistic cooperation between several fields of knowledge is required. Thus the science of soft robotics is understanding how and which scientific disciplines must be coupled to understand these soft robotic structures. This makes it challenging to develop and optimize designs, as the design space of the soft robots spans many coupled scientific disciplines. However, this also enables the emergence of exciting and novel behaviors that leverage the physics of the interactions between the brain, body and environment (Mengaldo et al., 2022). When compared to more ‘traditional’ robotics, where the power exchange from the environment to the robot can be compensated for in control, the development of a significantly different approach is required for soft systems, which naturally display a high level of underactuation. Therefore the development of soft robots calls for a new methodology of connecting different scientific disciplines which are traditionally treated independently.

### 1.2 Scientific motivation behind design of soft robots?

We propose that the scientific motivation behind soft robot design can be categorized into three different groups.

• Application solving, i.e. the development of new designs or soft robotic technologies that can extend the capabilities of robots to applications in which the compliance with the environment (Albu-Schäffer et al., 2007), robust disturbance rejection properties or energy efficiency are crucial (Kashiri et al., 2018). Examples include bio-medical instruments (Cianchetti et al., 2018), extreme environment exploration (Mahon et al., 2019) and safe human-robot interaction (Queißer et al., 2014).
• Advancing Theoretical Principles. Developing new theory or understanding that explains the behaviors or interactions of soft robotic elements with the environment. This could be driven by intrinsic curiosity opposed to being entirely application driven. Due to the potential for complex interactions between rigid and soft systems, there are many such problems in soft robotic research in the modeling and control domain.
• Improve our understanding of biological systems. Biological systems are typically, ‘soft’ or have soft components, thus developing bio-inspired or bio-mimetic systems allows us to further our understanding of the natural world through the creation of ‘robot-physics’ or similar approaches.

In many cases, the motivation straddles a number of these areas. Regardless of the specific motivation, to demonstrate significant scientific impact from soft robot designs, we must identify scientific problems or applications where the impact can be clearly shown, and is non-trivial. Previous work has motivated the need to analyze the significance of contributions (Hawkes et al., 2021), which could assist in enabling these key and impactful areas to be identified.

### 1.3 What makes soft robot design challenging?

The move towards the incorporation of soft and compliant structures in robots provides exciting new capabilities. However, several fundamental challenges need to be overcome to allow a significant improvement in soft robot design and manufacturing. The specific challenges include.

• Despite the recent efforts to include soft mechanisms and robotics within academic education, the transfer of knowledge on soft robot manufacturing, modeling and control, is still at its infancy. The interdisciplinary nature of soft robotics means that it is hard to be an expert across all scientific domains. This limits the ability to develop designs that simultaneously exploit material science, control theory, learning and fabrication. This challenge calls for education that highlight the connections between the different sub-fields and scientific disciplines. In addition, there is also a need for a common currency or language between these disciplines, to allow scientist from different training to meaningfully discuss and collaborate.
• Limited simulation and analysis tools. The non-linearity and properties of the materials and the high degrees of freedom means that simulation and analysis tools are limited, or do not have the precision, that is, available for rigid robotic systems.
• Lack of standardized or modular parts. There are few off-the-shelf components that can be purchased; an equivalent in the domain of ‘rigid’ robot systems would be smart servos which have integrated actuation and position/velocity control vastly simplifying actuation and control. This leads to comparatively slow fabrication which limits real world design exploration.

These challenges highlight the need for new approaches and tools for the construction of robots. In the following section we review some of the underlying approaches and the state-of-the art in soft robot design, discussing their advantages and limitations. Following this we present new technologies and design methodologies which could address some of the existing limitations.

## 2 Approaches to soft robot design

In this Section, we identify three main approaches currently, shown in Figure 3, used in the research community to design novel soft robots and discuss the relative merits of each.

### 2.1 Bio-inspiration and bio-mimetics

**FIGURE 2:**
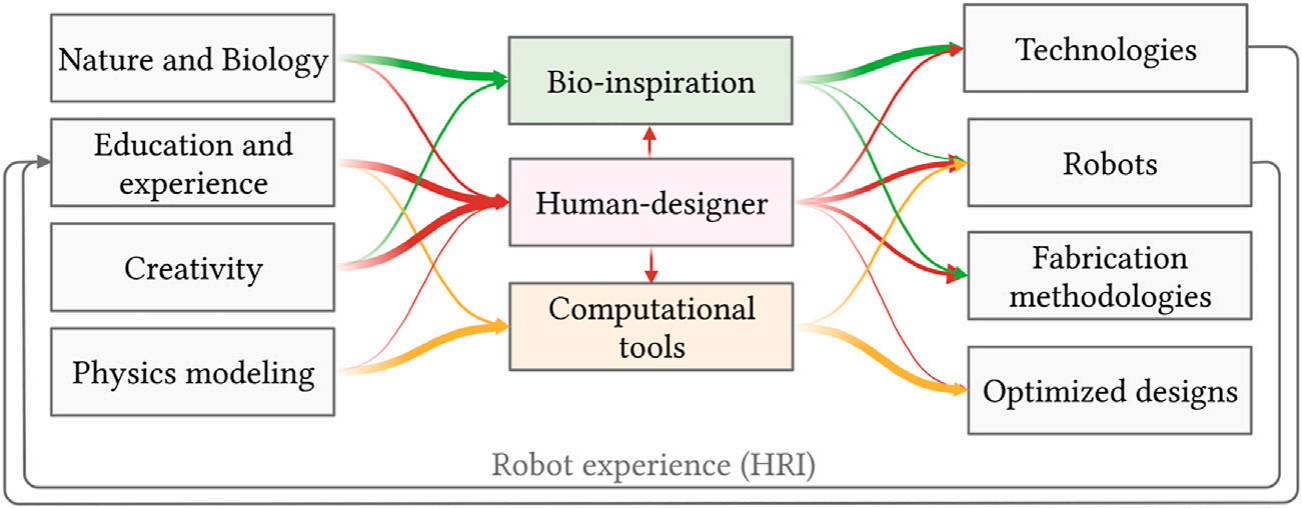
Schematic representation of the pipelines used for robot design. On the left, the pool of methods and fields which constitute the inspiration for robotics designs, in the center, the design approach used, and on the right the outcome of the design process. The magnitude of the arrows highlights the streams of information for the different in the pipelines, represented with arrows of different colours and intensities.

Nature provides an extraordinary number of examples of the enhanced motor capabilities generated by the introduction of compliant elements in the body structure. With the aim of replicating the capabilities of biological system, the research community put significant efforts into the design of robot inspired by nature (Kim et al., 2013; Kovač, 2014). Biological systems can inspire the design of soft robots in two main ways, namely bio-mimetics and bio-inspiration. Bio-mimetics seeks to reproduce the capabilities of natural systems through copying motions, appearance, and behaviors. Bio-inspiration instead looks at the founding physical principle in natural phenomena, to translate it into applications eventually far from the natural example. As presented in Figure 2, in the process of developing both bio-inspired and bio-mimetic systems, new technologies are created, novel combinations of existing technologies are proposed, new robotic structures are presented or novel fabrication methodologies are developed.

**FIGURE 3:**
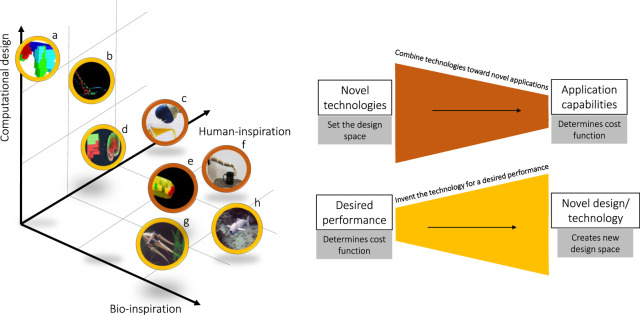
Pictorial representation of notable soft robotic design examples. Based on the methodology and process used, the design are placed in a three dimensional space spanning bio-inspired, computational and human-driven methodologies. As a fourth degree of classification, the color of the border indicates whether the design process was application oriented,—in red - or exploration oriented—in yellow. In the latter, the goal of building a robot with specific capabilities spurs the development of novel technologies with any of the pipelines presented in Figure 2. a-(Cheney et al., 2014), b-(Obayashi et al., 2022b), c-(Amend et al., 2012), d-(Kriegman et al., 2020), e-(Hawkes et al., 2017), f-(Cheng et al., 2012), g-(Obayashi et al., 2022a), h-(Katzschmann et al., 2018).

Mimicking nature allows us to exploit what has been learnt or identified through evolution. Hence we can copy mechanisms that are highly adapted for interactions with a specific environment. For example, fish display an incredible efficiency when moving in a water medium (Katzschmann et al., 2018), and human hands show incredible dexterity in grasping and manipulation tasks (Bicchi; 2000). Biological examples can be seen as the result of an ongoing evolutionary search taking place in the real world for millions of years. This perspective informs the designer in two ways.

• Nature already shows the optimal design for real tasks, which can hardly be found with human intuition or with a computationally-driven design optimization methods performed in simulation, due to the simulation to reality gap. However, it is not always clear what the real world objective functional for a given evolutionary process.
• Natural systems are able to perform both in artificial (e.g. human designed) and natural environments. Indeed, most of the artificial world has been engineered to interact ergonomically with natural systems. Therefore, robots that resemble natural structures, e.g. a human hand, can easily adapt to artificial environments, such as a door handle.

However, several drawbacks to bio-inspired robotics have to be noted. The design process (Kovač; 2014) to generate these new technological solutions often relies significantly on the designer expertise. Furthermore, when a biomimicry approach is used, the resulting designs are limited to natural examples. Moreover, while a biomimicry based approach could spur the designer’s creativity, there are substantial differences between living organisms and robots. First, the fitness function for which nature optimizes is not necessarily the optimal cost function for a robot. Indeed, some metrics that may be critical for biological entities, such as energy saving and optimal feeding tactics (Darwin, 1882) may be negligible for a robotic design. Similarly, when designing a robotic structure, elements such as manufacturing and scalability should be considered while they are not applicable to biological systems. Moreover, the characteristics of engineered components and natural structures are significantly different. While the building blocks for a natural system are cells, traditionally the roboticist has had to rely on rigid motors, electrical power supplies and a limited set of materials (Rus and Tolley; 2015). Another interesting example of the difference between natural and engineered systems regards the distribution of intelligence. It has been noted by Pfeifer et al. (2007) that nature is characterized by a high level of embodied intelligence. This decentralized distribution of intelligence is commonly justified by the low speed of transmission of sensory inputs and limited computational power in natural systems. While significant efforts have been placed to include this characteristic in robotic designs, this feature has not been crucial for robots, which can rely on high computational power and faster connection between sensors than natural systems.

### 2.2 Computational design

Computational design methods provide a framework to optimize the design parameters with respect to a specified fitness function. The design process is formalized as an optimization problem where the desired behavior is expressed by a cost function, evaluated on a set of variables which span the design space (Ha et al., 2018). The optimization is commonly performed in a simulated environment, which allows to evaluate the cost function over the design spaces. Note that the design spaces can span different morphologies, control strategies or a combination of the two.

With this methodology, the resulting design can significantly vary from natural examples and is not limited by the human intuition (Zhao et al., 2020; Hiller and Lipson, 2012). It therefore has the potential of generating particularly unexpected design solutions. In particular, it resulted to generate innovative results in problems with large design spaces and good simulation accuracy. At the same time, as presented in Figure 2, computational designs outperform human intuition in problems in which the goal is to optimize concepts that have clear mathematical descriptions but are not intuitively understood by humans (Stolpe; 2016).

However, the designer must typically specify the fitness function in clear mathematical terms. This has two main drawbacks. First, it can be hard or impossible to analytically specify a fitness function that captures the desired high-level behavior. Moreover, the solution of the optimization problem could be overly specific for the specified cost function and simulation environment, lacking robustness for slight changes in the environment and in the desired performance. Indeed, as the optimization is traditionally performed in simulation, this methodology is fundamentally limited by the simulation to reality gap. While the potential of simulations to generate solutions for robot designs in complex scenarios has been demonstrated for rigid robots, the simulation to reality gap is too wide to apply similar methods to soft structures (Hwangbo et al., 2019). This is mainly due to the difficulty of the simulation to capture the nonlinear material characteristics. Recently it has been shown that using a low fidelity simulation, tuned with feedback from a few real world experiments, may lead to better results for both classical rigid robots (Truong et al., 2022) and soft systems (Obayashi et al., 2022a). Another approach used to avoid the simulation to reality gap is performing the evolution of the control strategy (Veenstra et al., 2018) and of the robot morphology directly on the real system. Finally, it is hard to define a design space which takes into account fabrication possibilities, with the risk of optimizing for designs that are not physically plausible. Due to these challenges, successful translation of the computational designs to reality has been limited to few notable cases (Bächer et al., 2021; Bern et al., 2017; Blackiston et al., 2021; Sui et al., 2019).

### 2.3 Human-driven design

To this date, most novel designs have been generated by human intuition. When analyzing any design process, it is therefore pivotal to evaluate the role of the human in the design loop. First, the human expertise plays a clear role in both bio-inspired and computational design approaches. When following a bio-inspired pipeline (Kovač; 2014), the human designer has to translate the features observed in nature into a functional robotic system, therefore dealing with the fabrication constraints and with the challenges of modeling and controlling the system. In computational design, the role of the human is once again crucial, as the optimal result is highly dependent on the cost function and parameters selected by the human programmer. However, the design processes observed in humans are not limited to bio-inspiration and computational designs. Humans excel in finding connections between different experiences. In the context of robot design, humans are able to connect concepts from education, exploratory research and expertise in mechanical design, to build design intuition. It is possible also observe designs that are largely driven by human intuition and experience in mechatronic systems, such as the universal gripper (Amend et al., 2012).

As highlighted in Figure 2, the human-driven design is the only methodology in which the experience and interaction with existing soft robots is taken into account. This interaction provides the designer with new ideas on how to design mechatronic systems, control schemes, and fabrication methodologies in the next iteration of design. In this sense, each interaction with soft systems shapes the next generation of soft robots. Therefore, the human perception of the robotic systems (Jørgensen, 2022), and therefore features such as aesthetics and naturalness (Jørgensen et al., 2022) become crucial in the design of soft systems.

This prospective consequently inform us that human-driven design is highly dependent on the designer’s experience and expertise. Unfortunately, it takes a significant amount of effort and time for humans to become good designers. The learning process, i.e. the creation of a large design set, can be supported with an education system in which successful designs and tools are taught. To simplify the challenge of soft robotic design, it is crucial to develop well-characterized modular systems that can be combined in complex robotic structures. This modular approach to soft robotics would follow the approach of rigid robotics, which, to date, can rely on off-the-shelf motors, power supplies and structural components.

## 3 Advancing the science of soft robot design: Technology drivers and new methods

**FIGURE 4:**
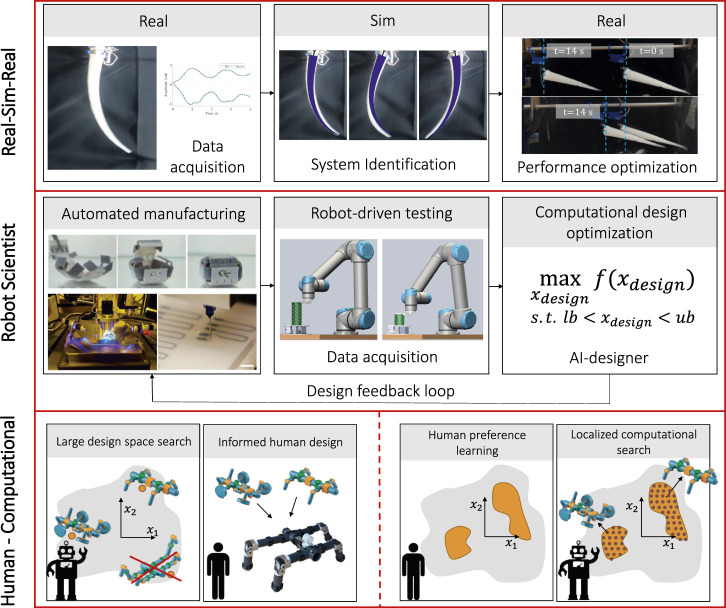
Schematic representation of the methodological advancements proposed. On the top row, first an embodied setup of the system to optimize is manufactured, and data about the behavior within the environment are captured with few experiments. The simulation is then informed of the real behavior through relity to simulation regression and identification methods. Finally, in simulation the structure morphology and control strategy is optimized, and brought back to reality. Thanks to the generality of the learnt model, the simulation can be used to optimize both the morphology and the control policy of the system (Stella et al., 2022). In the middle row, the robot is automatically manufactured, thanks to multi-material 3D printing, 4D printing or other automatic manufacturing technologies. The robot is then evaluated and tested by a second robotic system—the robot scientist—which captures data about the behavior. These real-world data inform the optimization algorithm, which generates the next iteration in the design by varying the robot structure, the control policy or a combination of the two. In the bottom row, two possible pipelines for human—computational design collaboration are highlighted. On the right, a large design space spanning different morphologies and control policies is explored computationally, and the best performing robot designs are returned to the human, which can then combine the most interesting features and account for manufacturability. On the left, the human informs the computational design algorithm on interesting zones of the design space, and on these the optimization method performs a extensive search for the best parameters.

To overcome some of the limitations of these methods, both technological development and new methodological approaches are needed. In this section we discuss how developments in technology could change the landscape of soft robot design. Additionally, we discuss some alternative approaches to robot design methodology, pictorially depicted in Figure 4, that go beyond the three presented.

### 3.1 Technology development

#### 3.1.1 Simulation & modeling tools

Improvements in simulation and modeling tools that can better capture both the physical interactions between robots and the environment, and soft robot systems would offer significant improvements for computational design. This could significantly reduce the simulation to reality gap and fabrication gap (Kriegman et al., 2020). Current advances and focuses on differentiable simulation have demonstrated the potential they offer in terms of optimization (Bächer et al., 2021; Bern et al., 2020). However, there remains challenges for these, for example, for modelling contact for soft robot manipulation. Advances in simulation tools could reduce the amount of data or processing required for system identification, and in the long term, fully eliminate the simulation to reality gap. Work on rigid robotic systems has shown the potential for simulation to reality transfer of learnt controllers, however we still require simulation tools for soft robotics where the required precision can be achieved at a reasonable ‘cost’.

A secondary role in which to exploit simulation and modeling tools is to identify the needs and requirements for technologies currently not available. For example, by identifying what properties of sensors or actuators are required to enable resulting output behaviors. This could help direct the requirements for new sensor, actuation or material technologies in a structured and formalized way.

#### 3.1.2 Physically adaptive and self-X technologies

Actuation, sensing or materials capabilities that offer physical adaption, or Self-X behaviors such as self-healing (Terryn et al., 2017), self-adaptation or self-growing could enable the morphology or robot properties to be optimized online (Vicari et al., 2022). This could allow for real-time adaption or exploration of the design space to find the optimal solution (Shah et al., 2021). Such technologies could limit the number of design iterations, that is, required for a single robot by leveraging the capabilities of the robots to self-assemble, self-structure of adapt. This may lead to the requirement of more bio-inspired approaches to design, for example, design through regeneration or developmental processes, such as 4D printing. The self-X capabilities offered by bio-hybrid technologies could be one way to explore such technologies (Guix et al., 2021).

#### 3.1.3 Robot ‘genes’: Building blocks

Development of ‘cell’ inspired building blocks with standardized manufacturing techniques, and accompanying mathematical description for modelling, could empower computational design searches. Such approaches could leverage biological concepts such as cell specialization and also co-ordination to enable ‘simple’ building blocks and which can combine together to achieve complex emergent behaviors at the organism level. The development of these ‘building blocks’ could accelerate fabrication and design implementation, which could be further assisted by dedicated simulation tools. The formulation of these into openly available tools could also improve the accessibility of soft robotics to other research domains.

### 3.2 Methodological advancements

In addition to improvements in technologies, there are also methodological advancements. Whilst these may be driven by the availability of new technologies and approaches, they can also result from new approaches or philosophies surrounding soft robot design.

#### 3.2.1 Real-sim-real

Transfer from simulation to reality directly is challenging. To leverage the advantages of simulation (Milana et al., 2021) whilst reducing the challenge of translating simulation to reality, we can instead start from reality, utilize system identification or other methods to capture the design space before returning to reality. Examples of this approach include using computer vision to extract the behavior of soft tentacles robot to generate a model which include information on the soft structure in the context of the environment, before controller optimization can be performed and then transferred back to reality (Stella et al., 2022). Iterative approaches of cycles or real-sim-real have also shown potential (Du et al., 2021). This approach could be further extended and formalized to allow for ‘capturing’ of the design space from real world robots, and also to mitigate or minimize challenges related to the simulation to reality gap.

#### 3.2.2 Real world evolution: Robot scientists

Repeated and automated experimentation in the real world removes the challenging of crossing the reality gap and also the fabrication gap. Robotic automation of the fabrication, testing and evaluation can remove the ‘human’ cost, and allow the tens, hundreds, or thousands of real-world evaluations that may be required. This has been demonstrated for more rigid systems (Brodbeck et al., 2015). Advances in robotic automation technologies and in fabrication of soft robots begin to make this possible for softer systems. Examples thus far include the optimization of the aerofoil of paper planes (Obayashi et al., 2022b) and modular soft walkers. By combining this approach with technologies which show physical adaption or self-X, this could simplify the real world fabrication through growth, self-assembly or other approaches. As such, this could make large scale testing and robot-driven evaluation increasingly feasible.

#### 3.2.3 Computational design tools for human assisted robot design

Computational design offers many advances over humans in their ability to analytically compute and explore and optimize a design space, especially for mathematically well-defined tasks. However, it lacks the intuition and creativity, that is, found in human designers. By developing computational tools that can be used by humans, for example, to predict the behavior of human driven design, or to narrow down a design space, the advances of both human and computational design can be leveraged. Learning-based tools to make suggestions to human operators have shown success in a number of different research areas (Christiano et al., 2017) and that could be applied to soft robot design.

This approach opens up questions as to the best mode of operation between human and robot designers. Exploring how the order or methodology of design affects the results should be further explored. Should computational or automated design should become before or after human design inspiration? Should these approaches be combined for an iterative approach? Within these approaches how can the creativity of designs be maintained?

#### 3.2.4 Community education

Human design will continue to significantly contribute to soft robot design. Thus, improving the education and skills of human designers would improve the designs generated by the humans that are central to the process. The interdisciplinary nature of soft robotics often means the education of researchers in this area is fragmented; for example, we have control specialists, that may lack the understanding of the properties of the materials constituting soft structures, or design specialists that do not understand the challenges of sensing technologies required to enable the control of such robots. Improving the interdisciplinary education of soft robotics to ensure that there is sufficient literacy across the necessary subfields of soft robotics could fundamentally improve the designs that are generated by human designers. Similarly, creating platforms (Bongard et al., 2012) in which researchers from different backgrounds can collaborate in an efficient way could enhance the variety and diversity of soft designs.

## 4 Discussion & conclusion

The potential offered by soft robotic technologies and components is significant; soft robotic technologies will clearly play a central role in shaping the future directions of robotics. This shift is going to require the development of new technologies and methodologies for exploring the design space of soft robot designs. Although this could be considered a challenge or limiting factor, it is also an opportunity to rethink and reapproach robot design. This could include a move towards reducing the separation between ‘rigid’ and ‘soft’ robots, but considering this as continuum, which incorporates robots of varying stiffness and softness. This wider design space will allow for us to explore a number of design approaches which are enabled by the technologies of soft robots, e.g. growing, adaptive or even bio-hybrid. These technologies can be combined with new methodologies to explore and generate new and exciting possibilities for soft robots. This will ultimately not only provide improvement performance in robotic systems, but also an improved understanding of why and how we build robots, advancing the science of soft robotic design.

## Data Availability

The original contributions presented in the study are included in the article/supplementary material, further inquiries can be directed to the corresponding author.
